# BEYOND THE MARGINS: INTIMATE PARTNER VIOLENCE AND TRANSGENDER OLDER ADULTS’ HEALTH AND WELL-BEING

**DOI:** 10.1093/geroni/igae098.2192

**Published:** 2024-12-31

**Authors:** Charles Hoy-Ellis, Hailey Jung, Loree Cook-Daniels, Hyun-Jun Kim, Karen Fredriksen-Goldsen

**Affiliations:** Goldsen Institute, University of Washington School of Social Work, Seattle, Washington, United States; University of Washington, Seattle, Washington, United States; FORGE, Milwaukee, Wisconsin, United States; University of Washington, Seattle, Washington, United States; University of Washington, Seattle, Washington, United States

## Abstract

Transgender older adults experience disproportionate violence and abuse, increasing their risk for deteriorating physical health, disability, depressive symptoms, and elevated stress levels when compared with their non-transgender age-peers. Studies have shown that barriers like fear of seeking healthcare, insufficient physical activity, internalized stigma, experiences of victimization, and a lack of social support play indirect roles in negatively impacting the health of this group, which has historically faced marginalization and neglect. According to the Health Equity Promotion Model, stigma and victimization significantly impact the mental health of older transgender adults. This study seeks to explore the connections between intimate partner violence (IPV), victimization, social resources, and mental health outcomes within this population. By analyzing cross-sectional data from a national survey involving lesbian, gay, bisexual, and transgender adults aged 50 to 95 years (N = 2,560), we found that transgender respondents (n = 167) experienced IPV at over double the rate of their non-transgender lesbian, gay, and bisexual counterparts (16% vs. 7%). Among transgender individuals, IPV strongly correlates with increased victimization and internalized stigma. IPV significantly predicted mental health quality of life and depressive symptomatology, independent of lifetime discrimination and victimization. Additional significant predictors of mental health outcomes were social network and support. Given the recent and continuing policy shifts nationwide affecting the transgender community, initiatives aimed at stigma reduction, victimization prevention, and bolstering social support are crucial for improving the health and overall well-being of transgender older adults. Insights from this research can guide stakeholders in countering regressive policies and legislation.

